# A randomised controlled trial of an exercise plus behaviour change intervention in people with multiple sclerosis: the step it up study protocol

**DOI:** 10.1186/s12883-014-0241-9

**Published:** 2014-12-21

**Authors:** Susan Coote, Stephen Gallagher, Rachel Msetfi, Aidan Larkin, John Newell, Robert W Motl, Sara Hayes

**Affiliations:** Department of Clinical Therapies, University of Limerick, Limerick, Ireland; Department of Psychology, Centre for Social Issues Research, University of Limerick, Limerick, Ireland; Multiple Sclerosis Society of Ireland, Dublin, Ireland; HRB Clinical Research Facility and School of Mathematics, Statistics and Applied Mathematics, National University of Ireland, Galway, Ireland; Department of Kinesiology and Community Health, University of Illinois, Urbana-Champaign, USA

**Keywords:** Exercise, Physical activity, Social cognitive theory, Behaviour change, Physiotherapy

## Abstract

**Background:**

Exercise has consistently yielded short-term, positive effects on health outcomes in people with multiple sclerosis (MS). However, these effects have not been maintained in the long-term. Behaviour change interventions aim to promote long-term positive lifestyle change. This study, namely, “Step it Up” will compare the effect of an exercise plus Social Cognitive Theory (SCT)-based behaviour change intervention with an exercise plus control education intervention on walking mobility among people with MS.

**Methods/design:**

People with a diagnosis of MS who walk independently, score of 0–3 on the Patient Determined Disease Steps, who have not experienced an MS relapse or change in their MS medication in the last 12 weeks and who are physically inactive will be randomised to one of two study conditions. The experimental group will undergo a 10-week exercise plus SCT-based behavioural change intervention. The control group will undergo a 10-week exercise plus education intervention to control for contact. Participants will be assessed at weeks 1, 12, 24 and 36. The primary outcome will be walking mobility. Secondary outcomes will include: aerobic capacity, lower extremity muscle strength, participant adherence to the exercise programme, self-report exercise intensity, self-report enjoyment of exercise, exercise self-efficacy, outcome expectations for exercise, goal-setting for exercise, perceived benefits and barriers to exercise, perceptions of social support, physical and psychological impact of MS and fatigue. A qualitative evaluation of Step it Up will be completed among participants post-intervention.

**Discussion:**

This randomised controlled trial will examine the effectiveness of an exercise plus SCT-based behaviour change intervention on walking mobility among people with MS. To this end, Step it Up will serve to inform future directions of research and clinical practice with regard to sustainable exercise interventions for people with MS.

**Trial registration:**

ClinicalTrials.gov, NCT02301442

## Background

Multiple Sclerosis (MS) is a chronic, often progressive disease of the central nervous system that results in a variety of impairments that cause limitations in activities and participation restrictions. Mobility limitations are common [1,2] even in the early stages of the disease [3] and are of significant concern to people with MS [4,5]. Despite recent advances in pharmacological treatments, exercise therapy remains the cornerstone of the management of mobility limitations among people with MS.

There is an expanding body of evidence suggesting that exercise has positive effects on many of the impairments and subsequent activity limitations and restrictions in participation for people with MS. Systematic reviews and meta-analyses demonstrate that exercise has a positive effect on muscle strength [6,7], aerobic capacity [7], mobility [1], quality of life [8,9] and fatigue [10,11]. One recent review confirmed the safety of exercise for people with MS [12]. However, despite increasing evidence for the beneficial effects of physical activity (PA) and exercise, there is consistent evidence that people with MS are, in fact, less active than their healthy counterparts [13] and those with other health conditions [14]. Klaren et al. (2013) [15] demonstrated that 20% of PwMS in their study met public health guidelines for mild-to-vigorous PA when compared to 47% of the healthy controls. This is of concern given that reduced PA levels are associated with reduced quality of life [16], and increased risk of cardiovascular disease [17]. Indeed, a recent population study found that people with MS have a 2.4 fold increased risk of death due to cardiovascular disease than the general population [18] and this may be linked with physical inactivity [19].

A recent multi-centre block randomised controlled trial, which evaluated the effectiveness of community exercise interventions for people with MS who had minimal gait impairment, demonstrated significant benefits of exercise in the community [20]. Physiotherapist- and fitness- instructor- led interventions consisted of combined aerobic and strengthening training components. That exercise intervention resulted in significant improvements in the physical impact of MS, psychological impact of MS, impact of fatigue and walking endurance [20]. Of note, however, is that these improvements were not maintained over the longer term and the effect on the primary outcome measure (walking mobility) was no longer significant 12 weeks post-treatment [20]. Moreover, there was significant attrition with 50% loss to follow up at 24 weeks [20]. It is therefore imperative that future research interventions facilitate long-term adherence and positive changes in PA behaviour, in order to inform the implementation of sustainable exercise interventions for people with MS.

A behavioural outcome of paramount importance for optimised, long-term effectiveness of exercise programmes is exercise-adherence, particularly post-intervention PA behaviour [21].

Social Cognitive Theory (SCT) is one of the most widely-adopted theoretical frameworks for understanding and optimising PA and other health behaviours. Along these lines, researchers have developed a programme of work investigating a SCT-based behaviour change intervention. The intervention involves workshops that aim to enhance exercise self-efficacy and focus on the provision of information relative to PA participation based on the principles of SCT; namely outcome expectations, self-efficacy, impediments and goal setting. Previous research has demonstrated that individuals who completed the SCT-based intervention attended more exercise sessions than individuals in the standard care group [22]. Subsequent research using an online delivery [23] observed significant positive effects on PA as measured by the Godin Leisure Time Exercise Questionnaire that were subsequently replicated as a significant, large increase on accelerometer step counts [24]. Supplementing the programme with video coaching further increased PA and this change was maintained 12 weeks post-intervention [25].

Given the positive effects of these two separate but complementary areas of work it is probable that a combination of exercise and SCT-based behaviour change approaches could further improve and maintain health outcomes for people with MS. Step it Up will compare the effect of an exercise plus SCT-based behaviour change intervention with an exercise plus control education intervention on walking mobility among people with MS. Our hypothesis is that those in the exercise and SCT-based intervention will achieve significantly more improvement in walking mobility than the control group post-intervention (12 weeks) and that this improvement will be maintained at 24- and 36- week follow up.

## Methods/design

### Study design

This will be a multi-centre, double blind, randomised, controlled trial comparing an exercise plus SCT-based intervention with an exercise plus contact control education intervention. Outcomes measures will be administered at weeks 1, 12, 24 and 36. The study will be performed in agreement with the Declaration of Helsinki and is approved by the Health Service Executive Mid-West Research Ethics Committee, the Galway University Hospitals Clinical Research Ethics Committee and the University of Limerick, Faculty of Education and Health Sciences Research Ethics Committee.

### Sample size

It is assumed that the effect of the intervention yields an average improvement in 6 Minute Walk Test (6MWT) distance of 36 m with an estimated standard deviation of 48.2 m [26]. In order to have 80% power (at the 5% significance level) to detect such a difference in mean improvement in 6MWT over the study period between groups, a sample of size 62 randomised equally to two arms (i.e. 31 per arm) is needed. In order to account for potential drop out a sample of size 72 will be recruited.

### Recruitment and eligibility

The participants will be recruited using the social media, email and postal communications of the MS Society of Ireland. Additionally people with MS will be recruited via neurology clinics in three urban locations in the west and south of Ireland. People who are interested in taking part in the trial will be invited to contact the research team at the University of Limerick via phone or email, wherein they will be given an opportunity to ask questions about Step it Up. When contacted, the researcher will explain the study in detail. Potential participants will be screened for selection criteria over the telephone and participant information leaflet and informed consent forms via post or email will be sent to each potential participant. Inclusion criteria are: (1) physician-confirmed formal diagnosis of MS, (2) aged 18 years or more, (3) Patient Determined Disease Steps score of 0–3, (4) a sedentary lifestyle (<30 minutes of moderate to strenuous exercise one day or more per week over the last six months) and (5) willing to give written informed consent. Exclusion criteria are: (1) pregnancy, (2) MS relapse in the last 12 weeks and (3) changes to MS medication or steroid treatment in the last 12 weeks.

### Random allocation procedures

Participants will be randomly allocated individually to the exercise plus SCT-based intervention or the exercise plus contact control education intervention. The allocation sequence will be concealed from all study personnel until after data collection is complete. All participants will be informed that we are evaluating the effect of combining exercise and education, and as such will be blind to their group allocation.

### Intervention procedures

#### Exercise plus contact control education intervention

The control group will receive an exercise and a didactic control education component. The exercise intervention will be common to both groups, will include aerobic and strengthening components and will be delivered by physiotherapists who are trained in the delivery of this standardised exercise intervention. The aim of the exercise component is to progressively increase the intensity of both aerobic and strengthening activities to enable the participants to reach the recently published MS exercise guidelines [7]. The aerobic activity will be walking, the intensity of which will be monitored using step rate measured using the Yamax digiwalker pedometer* (which will be provided to all participants) and an exercise log to document duration of walking exercise and number of steps taken. It is widely accepted that 100 steps per minute equates to three MET or moderate intensity PA among various clinical populations [27,28]. Similar values have been established for people with MS where the mean step-rate threshold at three METs for mean heights was 99 for people with MS who had minimal walking impairment and 96 for those with MS who had mild-moderate walking impairment [29]. Thus the target walking exercise intensity for both groups in the current study will be a rate of 100 steps per minute. Participants will begin with 10 minutes of walking twice weekly at a rate of 100 steps/minute and increase incrementally in 5 minute intervals over 5 weeks until they reach the guideline of 30 minutes twice weekly [7].

The strengthening programme is based on a community-based exercise programme that has been evaluated previously [20] and will consist of ten exercises targeting major muscle groups for the upper and lower extremities using elastic resistance band. The intensity and duration of the strengthening component of the intervention will be progressed by increasing the number of repetitions and sets and changing the resistance of the elastic resistance band used for each strengthening exercise. Participants will begin with one set of 10–15 repetitions and gradually increase the number of sets, repetitions and level of resistance until they meet the target of two sets of each exercise twice weekly with sufficient resistance that they are failing on the 12th repetition. Over the 10-week programme participants will attend the group exercise class on six occasions, supplemented with a telephone coaching call in the weeks without classes (intervention weeks 4, 6, 7 and 9). These telephone calls will consist of direct questions about the frequency, intensity, type and duration of exercise they have completed and whether they have experienced any adverse events or relapses. Of note, these telephone calls will also be conducted at weeks 16, 20 and 36, after the 10-week intervention has been completed. After each of the group exercise classes the control group will receive an education session about topics unrelated to PA behaviour, e.g. diet, vitamin D, sleep, temperature and hydration, and immunisations and vaccinations.

#### Exercise plus SCT-based intervention

The exercise plus SCT-based intervention group will receive the same exercise intervention as the control group (as described in the previous section).This group will also receive a behaviour change intervention based on the principles of SCT. The SCT-based education sessions will be delivered after each exercise session by physiotherapists who are trained in this area and will incorporate the principle elements of SCT including self-efficacy, outcome expectations, impediments and goal-setting. Session 1 will consist of a discussion on the benefits of exercise for persons with MS, instructions for beginning an exercise program, education on outcome expectations (physical, social, self-evaluative and mental outcome expectations) of the Step it Up intervention and instructions on effective self-monitoring of one’s behaviour (use of exercise logs and pedometers). Session 2 will include group discussion and physiotherapy-led guidance on setting specific, measureable, adjustable, action-oriented, realistic and time-based exercise and PA goals. Participants will also complete group-based written assignments and document their exercise goals during this session. Session 3 will focus on the concept of self-efficacy, with emphasis on the sources of self-efficacy, namely: mastery accomplishments, social modelling, social persuasion and the interpretation of physiological states. Session 4 will consist of discussion and documentation of the barriers and facilitators of exercise (environmental, social, health and cognitive and behavioural). Session 5 will focus on the concept of long-term maintenance of a physically-active lifestyle. The last education session will involve a celebratory ceremony for the participants to commend them on their successful completion of the Step it Up programme. Beyond providing presentation notes, individual reflection and written exercises, group discussion on each of the principles of SCT, and providing on-going feedback on all aspects of PA behaviour, the program will include video files of people with MS discussing PA behaviour and their experiences of initiating and maintaining a physically-active lifestyle. The use of video files of people with MS is grounded in a central tenet of SCT- the promotion of self-efficacy via social modelling and social persuasion. On the weeks when the participants do not attend group sessions, they will receive a telephone coaching call from the physiotherapist. These coaching calls will consist of guided conversations that consider the components of SCT delivered in the previous session and a revision of other components. Of note, participants will also receive telephone coaching calls from the physiotherapists at weeks 16, 20 and 36 (after the 10-week programme has been completed) in order to revise the topics covered during the Step it Up programme and to act as a support for long-term maintenance of a physically-active lifestyle.

#### Ensuring the fidelity of the interventions

A manual of operating procedures will be provided to the physiotherapists and incorporated into training for the physiotherapists and provided to them for their use throughout their delivery of the Step it Up programme. The physiotherapists’ adherence to the Step it Up intervention protocol will be verified using ad-hoc video analysis of the intervention programme. Fidelity of the intervention dose will be further monitored by recording participant attendance for each session and participant exercise completion and intensity of exercise using exercise log books throughout the 10-week intervention.

#### Measures

Outcome measures will be conducted pre-intervention (week 1), post-intervention (week 12), and at 3-months (week 24) and 6- months (week 36) follow-up. The outcome measures for this study include those measures recommended by an expert consensus group on a core set of outcome measures for exercise trials in MS [30] and demonstrate acceptable reliability and validity coefficients among people with MS. The participants will be sent all of the self-report outcome measures via post to complete prior to their objective assessment meeting with the blinded assessor (SH). Participants will receive instruction on how to complete each self-report measure according to standardised instructions for each measure of outcome. The postdoctoral researcher (SH) will be blind to allocation and will conduct all objective assessments on the week preceding the start of the Step it Up intervention.

#### Screening measure

Potential participants will be screened for eligibility for this study using the PDDS scale [31]. The PDDS scale contains a single item for measuring self-reported neurological impairment on an ordinal level from zero (Normal) to eight (Bedridden). Scores from the PDDS are linearly and strongly related with physician-administered Expanded Disability Status Scale (EDSS) scores [31].

#### Demographic and clinical information

Participants will be asked to supply details regarding age, gender, level of formal education, time since diagnosis of MS, duration of symptoms of MS, falls history, exercise history, marital status and employment status. Additionally, a researcher formally training in the use of the Expanded Disability Status Scale (EDSS) (SH) will administer the EDSS to all participants at baseline in order to gain a descriptive variable of disability. The EDSS is a clinical outcome measure of MS disease progression and is commonly the standard that other outcome measures are compared against [32]. It consists of functional systems subscales and a total score which is an ordinal rating ranging from 0 (normal neurological status) to 10 (death due to MS).

#### Primary outcome

Consistent with the finding that people with MS report mobility limitations as their greatest concern [5], the primary outcome will be walking mobility. This will be measured by the Six Minute Walk Test (6MWT), Timed Up and Go test (TUG) and the Multiple Sclerosis Walking Scale-12 (MSWS-12) in order to capture walking endurance, walking speed and participant-reported limitations in walking.

The 6MWT will involve the participants being instructed to walk for six minutes as quickly and safely as possible [33] and the distance (m) covered will be recorded. The 6MWT has demonstrated excellent test-retest reliability and concurrent validity among people with mild to moderate MS [33].

For the TUG, the participants will be instructed to stand up from a chair, walk 3 m, then turn around walk back to the chair and sit down, as quickly and safely as possible. The participants’ performance will be timed and the mean score of three performances of the TUG will be used in analysis. TUG-cognitive involves adding a cognitive task (subtracting three from a random number between 20 and 100) while performing the TUG. Previous research demonstrates excellent test-retest reliability for people with mild MS (EDSS: 0–4) [34].

The MSWS-12 will be used to measure participants’ self-reported walking limitations due to MS during the previous two weeks. All items are measured on a likert scale ranging from 1 (not at all) to 5 (extremely). Psychometric testing of the MSWS-12 has demonstrated that it has excellent internal consistency [35,36], test-retest reliability [37] and concurrent validity [38].

#### Secondary outcomes

The 5 times sit to stand test [39] will be used to measure lower extremity muscle strength. The participants will be instructed to stand up and sit down as quickly as possible when rising from a chair. It has demonstrated excellent construct validity among people with MS [33]. The 5 times sit to stand test has also demonstrated moderate to excellent concurrent validity among people with MS who had mild to moderate disability [40].

Aerobic capacity will be measured among participants using the Modified Canadian Aerobic Fitness Test [41] (mCAFT). The mCAFT is a graded step test and can predict VO_2peak_ using a published regression equation for people with MS [42].

The Hospital Anxiety and Depression Scale (HADS) [43] is a 14-item self-report scale and will be used to measure anxiety and depression among participants. The HADS has been validated among people with MS, demonstrating acceptable sensitivity (90% and 89% for the depression subscale) and specificity (87% and 81% for the anxiety subscale) [44].

The Symbol Digit Modalities Test (SDMT) [45] will be used to measure processing speed. Participants will be presented with a series of nine geometric symbols, each paired with a different single digit number in a key at the top of the page. Participants will be instructed to provide the digit associated with each corresponding symbol and the score shall be the number correctly completed in 90 seconds. The SDMT is a commonly-used, validated assessment of cognitive function in individuals with MS [46].

PA will be measured using the Godin Leisure-Time Exercise Questionnaire (GLTEQ) [47] and the short-form of the International Physical Activity Questionnaire (IPAQ) [48]. Evidence for the validity of these measures in people with MS has been reported [49-51]. The GLTEQ is a self-administered assessment that has three items that measure the frequency of strenuous, moderate, and mild physical activities for periods of more than 15 min during a person’s free time over the previous week. The short-form of the IPAQ was designed for population surveillance of PA among adults and measures the frequency and duration of vigorous-intensity activities, moderate-intensity activities, walking and sitting during a 7-day period.

Additionally, the SenseWear Arm band (SWA) will be used as an objective estimate of PA using both mean daily step count and mean daily energy expenditure estimates over a 7-day period. The acceptable criterion validity of the mean daily energy expenditure (kilocalorie estimates) over a 7-day period of the SWA has been demonstrated among people with mild disability with MS [52].

Adherence to the exercise programme will be documented throughout the 10-week intervention via self-report exercise logs. These exercise logs will capture information regarding participant attendance at the exercise classes. In relation to the strengthening component of the exercise programme, the number of sets, repetitions and colour of resistance band used will be recorded for each exercise during each exercise session. Additionally, the duration of each walking exercise session and pedometer-measured step count will be recorded in the exercise logs. The logs will also record participants’ reported enjoyment of each exercise session using a likert scale ranging from one (not at all) to seven (very much) and their perceived rate of exertion during each exercise session using a likert scale ranging from 6 (no exertion at all) to 20 (maximal exertion) [53].

Five questionnaires will be implemented to measure SCT domains. These include the Exercise Self-Efficacy Scale (EXSE), Exercise Goal Setting (EGS) scale [54], Multidimensional Outcomes Expectations for Exercise Scale (MOEES) [55], the Social Provisions Scale (SPS) [56] and an exercise benefits and barriers questionnaire. These questionnaires have been validated and have been used in previous research on PA [23,57,58].

The Multiple Sclerosis Impact Scale 29 (MSIS-29) [59] is a measure of the physical and psychological impact of MS from the patient’s perspective. Excellent internal consistency for the physical and psychological subscales, respectively, and moderate to excellent concurrent validity for the physical and psychological subscales, respectively have been reported for the MSIS-29 [60].

The Modified Fatigue Impact Scale (MFIS) is a 21-item self-report questionnaire and will measure the impact of fatigue on physical, cognitive and psychosocial aspects functioning among participants. Excellent test-retest reliability [61], internal consistency [62] and concurrent validity [61] has been reported in MS populations.

#### Statistical analysis

Suitable numerical statistics and graphical summaries will be used to describe characteristics of the sample at baseline and to assess the validity of any distributional assumptions needed for the formal analysis. The flow of trial participants and the level of missing data for all outcomes will be documented and an analysis into the cause of missingness (if present) will be conducted. All losses to follow-up and dropouts will be accounted, and where possible, reasons documented. The primary analysis will compare differences in the primary response variable, namely the difference in mean steps between the two treatment arms across the 12 week time period. Several analyses will be performed to compare the change in the primary response variable across time and between groups while adjusting for baseline as appropriate. These will include linear mixed models for a continuous response with different covariance structures compared in order to best model the correlation structure within subjects across time. All tests of significance will be two-sided and conducted at an alpha = 0.05 level of statistical significance. A further analysis will be performed to compare the estimated effect of the intervention when imputing values for all missing data in order to investigate the assumptions relating to missingness, and the effect, if any, on the overall conclusion. Multiple imputation will be performed using a Predictive Model Based Method using chained equations where each missing value is replaced by 20 imputed values. The results of each ‘complete’ model (i.e. with imputed values) will be averaged using the Barnard-Rubin adjustment method.

#### Qualitative analysis

Qualitative study of participants’ experiences of the Step it Up programme will be completed at weeks 12 and 36. This study is to compliment the quantitative findings and aspects and is response to a recent call for researchers to include a qualitative component to intervention trials [63]. In brief, 24 participants who complete the Step it Up programme will take part in one to one semi-structured interviews with questioning focusing on what worked and did not work; interviews will be audio-recorded, transcribed and analysed using thematic analysis using a qualitative descriptive design [64,65]. The pertinent features underlying qualitative description are: purposive sampling; a semi-structured interview schedule with open-ended questions; content data analysis and; descriptive results, close to the dataset [64,65].

## Discussion

This RCT will examine the effectiveness of an exercise plus SCT-based behaviour change intervention on walking mobility among people with MS. The Step it Up programme addresses limitations of exercise trials in the MS population highlighted in the literature to date. Step it Up will combine the well-recognised behaviour change principles of SCT with an evidence-based exercise programme, employing a mixed-methods approach and a long-term follow-up period. In order to systematically examine the intervention effects on walking mobility the Step it Up programme will include an array of outcome measures that will be used as manipulation checks, i.e. lower extremity muscle strength, aerobic capacity, SCT domains. Additionally, the exercise component of the Step it Up programme will be informed by published evidence-informed PA guidelines for people with MS [7]. The Step it Up programme will build on the work of Coote and colleagues [20,66] and Motl and colleagues [25] by evaluating the combined effects of an evidence-based exercise programme with a behavioural change programme using a RCT design. To this end, Step it Up will serve to inform future directions of research and clinical practice with regard to sustainable exercise interventions for people with MS.

## Abbreviations

MS: Multiple sclerosis
PA: Physical activity
SCT: Social cognitive theory
RCT: Randomised controlled trial
